# Tunable Luminescence in Sb^3+^-Doped Cs_3_LnCl_6_ Perovskites for Wide-Coverage Emission and Anti-Counterfeiting Applications

**DOI:** 10.3390/nano15231790

**Published:** 2025-11-27

**Authors:** Lianao Zhang, Le Chen, Sai Xu, Yongze Cao, Xizhen Zhang, Hongquan Yu, Yuefeng Gao, Baojiu Chen

**Affiliations:** 1 School of Science, Dalian Maritime University, Dalian 116026, China; zla010701@dlmu.edu.cn (L.Z.); chenle0991@dlmu.edu.cn (L.C.); cyz@dlmu.edu.cn (Y.C.); zhangxizhen@dlmu.edu.cn (X.Z.); yuhq7808@dlmu.edu.cn (H.Y.); bjchen@dlmu.edu.cn (B.C.); 2 Marine Engineering College, Dalian Maritime University, Dalian 116026, China

**Keywords:** Sb^3+^ doping, 0D rare-earth-based metal halides, energy transfer, STE, optical anti-counterfeiting

## Abstract

Zero-dimensional (0D) rare-earth-based metal halides show great potential in photonic and optoelectronic applications owing to their high stability, strong exciton confinement, and tunable energy levels. However, the weak absorption and narrow 4f-4f transitions of rare-earth ions limit their performance. To address this, a series of Sb^3+^-doped Cs_3_LnCl_6_ (Ln: Yb, La, Eu, Ho, Ce, Er, Tb, Sm, Y) nanocrystals were synthesized via a hot-injection method to study the role of Sb^3+^ doping. Sb^3+^ incorporation induces strong broadband self-trapped exciton (STE) emission from Jahn–Teller-distorted [SbCl_6_]^3−^ units and enables efficient energy transfer from STEs to rare-earth ions. As a result, the photoluminescence intensity and spectral tunability are improved, accompanied by bandgap narrowing and enhanced light absorption. Different lanthanide hosts exhibit distinct luminescence behaviors: La-based materials show dominant STE emission, while Tb-, Er-, Yb-, Ho-, and Sm-based systems display STE-mediated energy transfer and enhanced f-f emission. In Eu- and Ce-based hosts, unique mechanisms involving Eu^2+^/Eu^3+^ conversion and Ce^3+^ → STE energy transfer are observed. Moreover, composition-dependent emissions in Sb^3+^-doped Cs_3_Tb/EuCl_6_ enable a dual-mode color and spectral encoding strategy for optical anti-counterfeiting. This study highlights the versatile role of Sb^3+^ in tuning electronic structures and energy transfer, offering new insights for designing high-performance rare-earth halide materials for advanced optoelectronic applications.

## 1. Introduction

In recent years, three-dimensional (3D) lead halide perovskites have achieved remarkable breakthroughs in photovoltaic efficiency, owing to their high absorption coefficients, long carrier diffusion lengths, and tunable bandgaps [1,2,3]. However, the intrinsic issues of toxicity and poor stability in lead-based perovskites severely hinder their practical applications [4,5]. Therefore, the development of lead-free, stable, and efficient perovskite materials has become a major research focus. Among the various alternatives, zero-dimensional (0D) rare-earth-based halide perovskites have attracted increasing interest because of their unique structural and optical characteristics [6]. Unlike 3D or 2D perovskites, the inorganic octahedra or clusters in 0D systems are completely isolated by organic cations or inert matrices, lacking long-range connectivity within the crystal lattice [7,8,9]. This not only enhances resistance to moisture, heat, and photo-induced degradation by suppressing ion migration and phase transitions commonly observed in 3D perovskites, but also promotes strong quantum confinement and exciton localization, which result in large exciton binding energies and highly efficient radiative recombination, thereby yielding high photoluminescence quantum yields (PLQYs) [10,11,12]. These distinctive characteristics endow 0D rare-earth halide perovskites with great potential in optoelectronic devices, scintillators, and sensors.

Nevertheless, the *f-f* transitions of rare-earth ions are characterized by small absorption cross-sections and narrow emission bands, which limit the applications of rare-earth-based metal halides in broadband tunable emission and efficient energy utilization [13,14]. Therefore, overcoming the intrinsic constraints of rare-earth emission through material design and extending their spectral range has become a current research focus [15]. Ion doping has been proven to be an effective approach to improve the optical performance of perovskites. In recent years, metal ions with an ns^2^ electronic configuration, such as Sb^3+^, Bi^3+^, and Te^4+^, have been extensively explored [16,17]. Among them, Sb^3+^ ions, with their characteristic 5s^2^ outer electron configuration, can introduce new electronic states into the host lattice, promote the formation of self-trapped excitons (STEs), and establish efficient energy transfer channels, making Sb^3+^ an ideal candidate for tailoring luminescence properties [18]. For example, Lin et al. found that Sb^3+^ doping in Cs_2_NaLuCl_6_ creates dopant-induced STEs that establish additional energy transfer channels to Er^3+^, significantly enhancing the transfer efficiency from 8% to 33%. Xia’s group reported that Sb^3+^ doping in Cs_2_InCl_5_·H_2_O induces strong broadband yellow emission with a high photoluminescence quantum yield of 95.5%. In addition, Lu’s group reported that Sb^3+^ doping in Cs_3_TbCl_6_ nanocrystals introduces [SbCl_6_]^3−^-induced STE that enhance energy transfer and photon absorption, greatly improving green emission intensity and luminescence efficiency [19,20,21]. This combined mechanism of broadband excitation and energy transfer offers a new pathway to overcome the intrinsic limitations of rare-earth luminescence.

Here, a series of Sb^3+^-doped Cs_3_LnCl_6_ nanocrystals were synthesized by thermal injection, and the effect of Sb^3+^ doping on the structural and optical properties of Cs_3_LnCl_6_ were systematically investigated. The incorporation of Sb^3+^ ions was found to preserve the 0D monoclinic phase while introducing Jahn–Teller distortion in [SbCl_6_]^3−^ octahedra, thereby generating strong broadband STE emission and forming efficient energy transfer pathways between STE states and lanthanide ions. Comparative optical analysis revealed distinct emission behaviors among different lanthanide hosts, including STE-dominated emission, STE-mediated f-f enhancement, charge-transfer-related processes, and energy transfer from Ce^3+^ to STEs. Furthermore, composition-dependent emissions observed in Sb^3+^-doped Cs_3_Tb/EuCl_6_ enabled the realization of dual-mode color and spectral encoding for optical anti-counterfeiting. These findings provide fundamental understanding of the multifunctional role of Sb^3+^ in tailoring electronic structures, regulating energy transfer dynamics, and enhancing emission tunability in rare-earth-based halide perovskites.

## 2. Experimental Section

### 2.1. Materials

Cesium acetate (Cs(OAc), 99%), lanthanum acetate (La(OAc)_3_, 99%), europium acetate (Eu(OAc)_3_, 99%), terbium acetate (Tb(OAc)_3_, 99%), erbium acetate (Er(OAc)_3_, 99%), holmium acetate (Ho(OAc)_3_, 99%), cerous acetate (Ce(OAc)_3_, 99%), yttrium acetate (Y(OAc)_3_, 99%), samarium acetate (Sm(OAc)_3_, 99%), ytterbium acetate (Yb(OAc)_3_, 99%), antimony acetate (Sb(OAc)_3_, 99%), oleic acid (90%), 1-octadecene (90%), oleylamine (90%), and chlorotrimethylsilane ((CH_3_)_3_SiCl, TMSCL, 99%) were purchased from Aladdin (Shanghai, China).

### 2.2. Synthesis of Sb^3+^-Doped Cs_3_LnCl_6_ Perovskite NCs

0.6 mmol Cs(OAc), 0.4 mmol Ln(OAc)_3_ (Ln: La, Ho, Sm, Eu, Tb, Er, Yb, Ce, Y), 0.004 mmol Sb(OAc)_3_, 2.5 mL oleic acid, 1 mL oleylamine, and 10 mL 1-octadecene were placed into a 100 mL three-necked flask, which was heated to 105 °C under vacuum for 60 min. Then, the reaction mixture was heated to 190 °C under a nitrogen atmosphere, and 0.5 mL TMSCL was rapidly injected. After two minutes of reaction, the mixture solution was immediately cooled to room temperature in an ice-water bath. The reaction mixture was then poured into a centrifuge tube and centrifuged at 10,000 rpm for 10 min. After removing the supernatant, the precipitate was washed with toluene three times. The obtained NCs were dispersed in toluene for future use.

### 2.3. Characterizations

The surface morphologies of the samples were characterized using an FEI Talos F200x (Hillsboro, OR, USA) transmission electron microscope (TEM). The crystal structure of the samples was analyzed by an X-ray diffractometer (XRD, Rigaku Dmax Ultima+, Tokyo, Japan) using Cu-Kα (λ = 1.54178 Å, 2°/min) as the radiation source. X-ray photoelectron spectroscopy (XPS) analysis was performed on a Thermo Scientific K-Alpha (East Grinstead, UK) spectrometer equipped with a monochromatic Al Kα X-ray source (hv = 1486.6 eV). The photoluminescence excitation (PLE) and photoluminescence (PL) spectra were collected using an Edinburgh spectrofluorometer FLS-1000 (Livingston, UK) equipped with an Xe 900 lamp as the excitation source. Diffuse reflectance spectra were obtained using a Shimadzu UV-3600 PC (Kyoto, Japan). Fluorescence decay curves were measured by an FLS-1000 using time-dependent single-photon counting (TCSPC) technology and an external EPL laser. The absolute PLQY was measured using an Edinburgh Instruments FLS1000 spectrofluorometer equipped with an integrating sphere. The excitation and emission spectra were recorded under identical conditions, and the absorbed and emitted photon numbers were obtained by comparing excitation profiles with/without the sample and integrating the corrected emission spectra, respectively. The quantum efficiency was then calculated as the ratio of emitted to absorbed photons.

## 3. Results and Discussion

**Figure 1:**
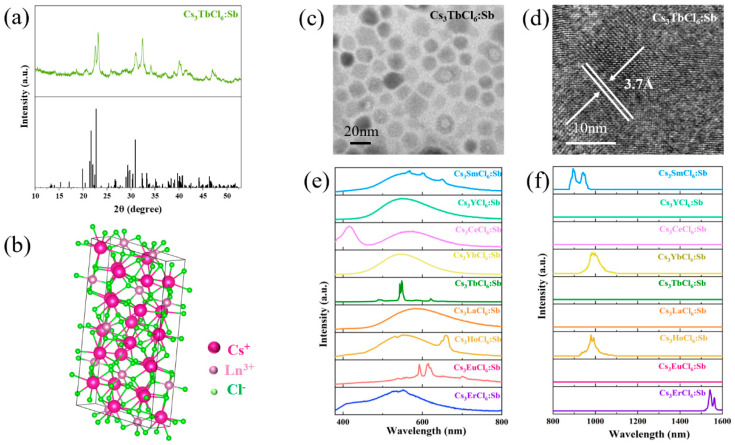
(**a**) XRD pattern of Cs_3_TbCl_6_:Sb^3+^ NCs. (**b**) Crystal structure model of Cs_3_LnCl_6_:Sb^3+^. (**c**) TEM and (**d**) HR-TEM images of Cs_3_TbCl_6_:Sb^3+^ NCs. PL spectra of Cs_3_LnCl_6_:Sb^3+^ NCs (Ln: Yb, La, Eu, Ho, Ce, Er, Tb, Sm, Y) in the (**e**) visible and (**f**) NIR regions.

Sb^3+^-doped Cs_3_LnCl_6_ (Ln: Yb, La, Eu, Ho, Ce, Er, Tb, Sm, Y) NCs were synthesized using a thermal injection method. The crystal structures of the samples were examined by XRD, as shown in Figure 1a and Figure S1a. Comparison with standard reference patterns (PDF#81-2480) of Cs_3_LaCl_6_ NCs confirms that Sb^3+^-doped Cs_3_LnCl_6_ NCs exhibit high crystallinity without impurity phases. In addition, the XRD pattern of undoped Cs_3_LaCl_3_ was measured (Figure S1b), which matches well with the standard reference card, indicating that Sb^3+^ doping does not affect the crystal structure of the samples. To further analyze the structural parameters, Rietveld refinement was performed on Cs_3_EuCl_6_:Sb^3+^ as an example (Figure S2a), and the corresponding crystal parameters and reliability factors are listed in Table S1. The refinement results reveal that Cs_3_EuCl_6_:Sb^3+^ NCs crystallize in a non-centrosymmetric zero-dimensional monoclinic structure with a C2/c space group. In this structure, each Ln^3+^ ion is coordinated with six surrounding Cl^−^ ions to form an isolated [LnCl_6_]^3−^ octahedral unit, while Cs^+^ ions occupy the interstitial sites [22], as illustrated in Figure 1b. TEM images in Figure 1c and Figure S1c–j show that the nanocrystals are cubic, uniformly distributed, and possess sizes ranging from 10 to 20 nm. The high-resolution TEM image in Figure 1d shows distinct lattice fringes with the lattice spacing of 3.7 Å for Cs_3_TbCl_6_:Sb^3+^. Moreover, the thermal stability of Cs_3_LaCl_6_:Sb^3+^ was evaluated using thermogravimetric analysis (Figure S2b). A slight mass loss below 100 °C is attributed to the removal of physically adsorbed moisture. As the temperature increases to approximately 300 °C, the observed weight reduction is likely associated with the decomposition or volatilization of surface-bound organic ligands such as oleic acid. Notably, even at 800 °C, the sample retains more than 80% of its initial mass, demonstrating the good thermal stability of the material.

**Figure 2:**
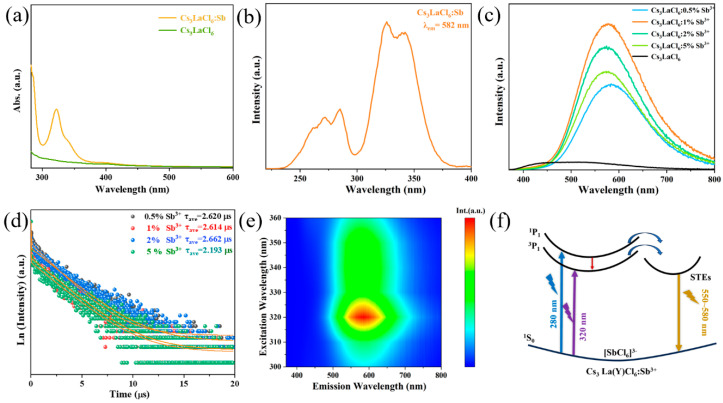
(**a**) Absorption spectra comparison of Cs_3_LaCl_6_:Sb^3+^ and Cs_3_LaCl_6_. (**b**) PLE spectrum of Cs_3_LaCl_6_:Sb^3+^ monitored at 582 nm. (**c**) PL spectra of Cs_3_LaCl_6_:Sb^3+^ NCs doped with different Sb^3+^. (**d**) The decay curves and corresponding fitting curves of Cs_3_LaCl_6_:Sb^3+^ NCs doped with different Sb^3+^. (**e**) Pseudo-color images of emission spectra under different excitations. (**f**) Schematic diagram of the luminescence mechanism of Cs_3_La(Y)Cl_6_:Sb^3+^ NCs.

Firstly, the luminescence spectra of Cs_3_LnCl_6_:Sb^3+^ NCs were investigated. As shown in Figure 1e,f, under excitation at 320 nm, the emission spectra of Cs_3_LnCl_6_:Sb^3+^ cover the visible to near-infrared region ranging from 380 to 1600 nm. In the visible region, Cs_3_LnCl_6_:Sb^3+^ NCs (Ln: Sm, Y, Ce, Yb, La, Ho, Er, Tb, Eu) exhibit broadband emission in the range of 370–800 nm. In addition, intrinsic emissions were also observed in several nanocrystals, such as Cs_3_SmCl_6_:Sb^3+^ showing emissions at 572 nm (^4^G_5/2_ → ^6^H_5/2_), 600 nm, (^4^G_5/2_ → ^6^H_7/2_) and 650 nm (^4^G_5/2_ → ^6^H_9/2_); Cs_3_CeCl_6_:Sb^3+^ at 410 nm (5d → ^2^F_7/2_); Cs_3_HoCl_6_:Sb^3+^ at 652 nm (^5^F_5_ → ^5^I_8_); Cs_3_ErCl_6_:Sb^3+^ at 550 nm (^4^S_3/2_ → ^4^I_15/2_); Cs_3_EuCl_6_:Sb^3+^ at 593 nm (^5^D_0_ → ^7^F_1_), 612 nm (^5^D_0_ → ^7^F_2_), and 703 nm (^5^D_0_ → ^7^F_4_); and Cs_3_TbCl_6_:Sb^3+^ at 493 nm (^5^D_4_→^7^F_3_), 549 nm (^5^D_4_ → ^7^F_4_), 589 nm (^5^D_4_ → ^7^F_5_), and 625 nm (^5^D_4_ → ^7^F_6_). In the infrared region, the corresponding intrinsic emissions can be observed at 893 nm (^4^G_5/2_ → ^6^F_3/2_) and 952 nm (^4^G_5/2_ → ^6^F_5/2_) for Cs_3_SmCl_6_:Sb^3+^; 980 nm (^2^F_5/2_ → ^2^F_7/2_) for Cs_3_YbCl_6_:Sb^3+^, 982 nm (^5^I_5_ → ^5^I_8_) for Cs_3_HoCl_6_:Sb^3+^; and 1550 nm (^4^I_13/2_ → ^4^I_15/2_) for Cs_3_ErCl_6_:Sb^3+^, respectively. To investigate the origin of the broadband emission, the absorption, photoluminescence excitation (PLE), photoluminescence (PL) spectra, and PL decay curves of Cs_3_LaCl_6_:Sb^3+^ were measured as a representative. As shown in Figure 2a, Cs_3_LaCl_6_ exhibit a weak absorption band around 400 nm. After doping with Sb^3+^, an absorption peak at 320 nm is observed, consistent with previous reports [23,24]. As illustrated in Figure 2b, the PLE spectra of Cs_3_LaCl_6_:Sb^3+^ monitored at 582 nm display two excitation bands within 250–300 nm and 300–360 nm, corresponding to the transitions of ^1^S_0_→^3^P_2_ and ^1^S_0_→^3^P_1_, respectively [25]. The asymmetry of the doublet state originates from the dynamic Jahn–Teller effect, a typical feature of ions possessing an ns^2^ outer electron configuration, including Te^4+^, Sb^3+^, and Bi^3+^ [26,27,28]. The PL spectra of Cs_3_LaCl_6_ with varying Sb^3+^ doping concentrations in Figure 2c show that Sb^3+^ doping induces a red shift in the emission center of Cs_3_LaCl_6_, accompanied by a pronounced enhancement in luminescence intensity. Moreover, the luminescence intensity gradually increases with Sb^3+^ doping, reaching its maximum at a doping concentration of 1%, where Cs_3_LaCl_6_:Sb^3+^ exhibits the highest photoluminescence quantum yield (PLQY) of 81.73%; however, in undoped Cs_3_LaCl_6_, the PLQY is only 38.02% [22] (Figure S3a). When the doping concentration exceeds 1%, the luminescence intensity decreases progressively. The luminescence decay curves of Cs_3_LaCl_6_ with different Sb^3+^ are recorded in Figure 2d. The decay profiles are well described by a biexponential function, where the fast and slow components (τ_1_ and τ_2_) correspond to the ^3^P_1_-^1^S_0_ and ^1^P_1_-^1^S_0_ transitions, respectively [29]. The fitted parameters are summarized in Table S2, showing that the average lifetime remains nearly unchanged as the Sb^3+^ concentration increases from 0.5% to 2%, but reduces to 2.193 µs when the Sb^3+^ concentration further increases to 5%. This decrease is attributed to enhanced non-radiative relaxation induced by high-concentration doping. To clarify the luminescence mechanism, excitation-wavelength-dependent emission spectra of Cs_3_LaCl_6_:Sb^3+^ were examined. In general, ion-related emission varies with energy transfer processes that depend on the excitation wavelength. As shown in Figure 2e, the PL spectra mapping of Cs_3_LaCl_6_:Sb^3+^ consistently displays a single emission center at 582 nm under different excitation wavelengths, thereby excluding the contribution of ionic emissions. It should be noted that defect-associated emissions typically produce broad spectral bands, with their intensity reaching saturation at elevated excitation power densities. To further verify this, power-dependent PL spectra of Cs_3_LaCl_6_:Sb^3+^ were collected (Figure S3b), demonstrating a continuous growth in emission intensity with increasing excitation power while maintaining an unchanged spectral profile. Consequently, the possibility of persistent defect emissions is excluded, as evidenced by the linear relationship between emission intensity and excitation power density (Figure S3c). Overall, the luminescence center of Cs_3_LaCl_6_ has a significant red shift after Sb^3+^doping, resulting in a large Stokes shift, wavelength-independent spectra, and microsecond-scale lifetime. these results provide clear evidence that the yellow broad emission is well correlated with self-trapped exciton (STE), which are driven by the Jahn–Teller distortion of the [SbCl_6_]^3−^ octahedra [30]. The schematic diagram of the luminescence process of Cs_3_LaCl_6_:Sb^3+^ is shown in Figure 2f. When excited by higher energy, electrons are excited from the ground state ^1^S_0_ to the higher excited state ^1^P_1_; some electrons undergo an intersystem crossing process from the singlet state (^1^P_1_) to the triplet state (^3^P_1_), and the rest of the electrons are transferred to the STE. When excited by lower energy, electrons are excited from the ground state ^1^S_0_ to the ^3^P_1_ excited state. Subsequently, the excited electrons are transferred to the STE and eventually return radiatively to the ^1^S_0_ ground state, giving rise to a broad emission band centered at 550 nm [31].

**Figure 3:**
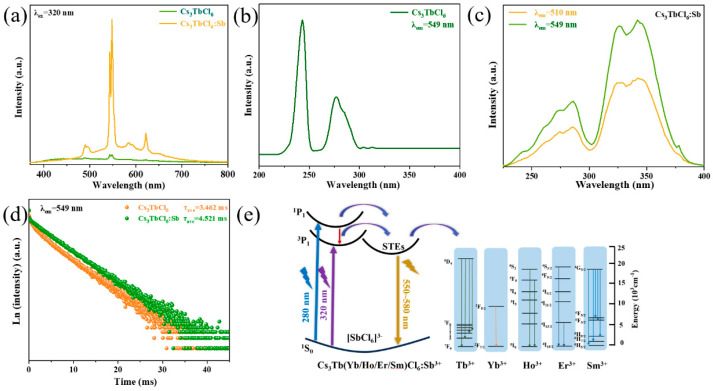
(**a**) PL spectra of Sb^3+^-doped and undoped Cs_3_TbCl_6_ NCs. (**b**) PLE spectra of Cs_3_TbCl_6_ NCs at 549 nm. (**c**) PLE spectra of Cs_3_TbCl_6_:Sb^3+^ NCs at 549 nm and 510 nm. (**d**) PL decay curves of Sb^3+^-doped and undoped Cs_3_TbCl_6_ NCs monitored at 549 nm. (**e**) Schematic diagram of the luminescence mechanism of Cs_3_Tb(Yb/Ho/Er/Sm)Cl_6_:Sb^3+^ NCs.

For Cs_3_LnCl_6_:Sb^3+^ (Ln = Tb^3+^, Er^3+^, Yb^3+^, Ho^3+^, Sm^3+^), the emission spectra display not only a broad emission band centered at 550 nm but also the sharp intrinsic narrow-band emissions of the rare-earth ions. Moreover, as shown in Figure 3a and Figure S4, Sb^3+^ doping markedly enhances the intrinsic emission intensity of the rare-earth ions (in the undoped samples, the infrared emission is too weak to be detected). As illustrated in Figure S5, the Sb^3+^-doped Cs_3_LnCl_6_ (Ln = Tb^3+^, Er^3+^, Yb^3+^, Ho^3+^, Sm^3+^) samples exhibit absorption peaks consistent with those of Cs_2_LaCl_6_:Sb^3+^. According to the Tauc plots derived from the absorption spectra, the bandgap of Cs_3_LnCl_6_ (Ln = Tb^3+^, Er^3+^, Yb^3+^, Ho^3+^, Sm^3+^) significantly narrows after Sb^3+^ incorporation (Figures S6 and S7). A narrower bandgap lowers the energy threshold required for photon absorption, thereby improving the absorption efficiency of the material [32]. To investigate the mechanism of narrow-band emission enhancement, the excitation spectra of Cs_3_TbCl_6_ and Cs_3_TbCl_6_:Sb^3+^ were examined. As shown in Figure 3b, for the 549 nm intrinsic emission of Cs_3_TbCl_6_, excitation peaks at 240 nm and 270 nm are observed, corresponding to the intrinsic excitation of Tb^3+^ and the Cl^−^→Tb^3+^ charge-transfer band, respectively [33]. After Sb^3+^ doping, however, the excitation spectrum monitored at 549 nm shows the disappearance of both intrinsic and charge-transfer excitation peaks of Tb^3+^, while broad excitation bands emerge in the ranges of 250–280 nm and 320–340 nm (Figure 3c). These broad features are identical to those observed at the 550 nm broadband emission and are similar to the excitation spectrum of Cs_3_LaCl_6_:Sb^3+^, suggesting that the enhancement of narrow-band emission originates from energy transfer from Sb^3+^-induced STEs. To further elucidate the enhancement mechanism, fluorescence decay curves of Cs_3_TbCl_6_ at 549 nm were measured before and after Sb^3+^ doping. The fitted results show that the Tb^3+^ lifetime increases from 3.462 ms to 4.521 ms (Figure 3d), implying that Sb^3+^ incorporation improves the crystal field environment, reduces structural defects in Cs_3_TbCl_6_, and suppresses non-radiative relaxation, thereby enhancing emission intensity [34], which is consistent with previously reported literature [20]. Subsequently, the excitation spectra of broadband and intrinsic infrared emissions were monitored for Cs_3_LnCl_6_:Sb^3+^ (Ln = Er^3+^, Yb^3+^, Ho^3+^, Sm^3+^). As shown in Figure S8, the excitation spectrum monitored at the intrinsic emission position exhibits a similar profile to that of the broadband emission, confirming that their intrinsic luminescence originates from STE-mediated energy transfer. Notably, in the excitation spectrum of Cs_3_YbCl_6_:Sb^3+^, the excitation peak intensity at 280–300 nm monitored at 992 nm is higher than that at 546 nm, which can be attributed to the superposition of STE excitation and the charge-transfer band of [YbCl_6_]^3−^. To further verify the energy transfer process, the broadband emission lifetimes of Cs_3_LnCl_6_:Sb^3+^ (Ln = Er^3+^, Yb^3+^, Ho^3+^, Sm^3+^, Y^3+^) were measured, yielding values of 16.033 ns, 906.122 ns, 93.826 ns, 155.381 ns, and 1382.209 ns (Figure S9), respectively. These results indicate that in Cs_3_LnCl_6_:Sb^3+^, which possesses the intrinsic emissions of rare-earth ions, the lifetimes of STE emission are all shorter than 1 μs, significantly shorter than those in Cs_3_YCl_6_:Sb^3+^ and Cs_3_LaCl_6_:Sb^3+^, providing indirect evidence for energy transfer from STE to Ln^3+^ ions (Ln = Er^3+^, Yb^3+^, Ho^3+^, Sm^3+^) [35]. Similar strategies have been reported in Cs_2_NaInCl_6_:Sb^3+^/Ln^3+^ [36]. The luminescence mechanism of Cs_3_LnCl_6_:Sb^3+^ (Ln = Tb^3+^, Er^3+^, Yb^3+^, Ho^3+^, Sm^3+^) is illustrated in Figure 3e. When excited at 280 nm, the electrons are excited from the ground state ^1^S_0_ to the higher ^1^P_1_ excited state, some electrons undergo an intersystem crossing process from ^1^P_1_ to ^3^P_1_, while the rest are transferred to the self-trapping state. Under excitation by 320 nm ultraviolet light, electrons are excited from the ground state of ^1^S_0_ to the excited state of ^3^P_1_ and then transferred to the STE. After that, a portion of electrons from the self-trapped state radiation transition to ^1^S_0_ ground state and emit broadband emission. The rest of the electrons are transferred to the excited state of rare-earth ions through the energy transfer process and then return to the ground state through radiation transition and emit narrow-band intrinsic emission (Table S3). In addition, analysis of the broadband emission spectra of Sb^3+^-doped Cs_3_LnCl_6_ reveals that the emission centers are not fixed but red-shift progressively with increasing ionic radius (Figure S10). This trend is attributed to the local asymmetry at the Sb^3+^ dopant sites within the zero-dimensional perovskite lattice. A larger ionic radius induces stronger local distortion, ultimately leading to the red-shift in the emission centers [37].

**Figure 4:**
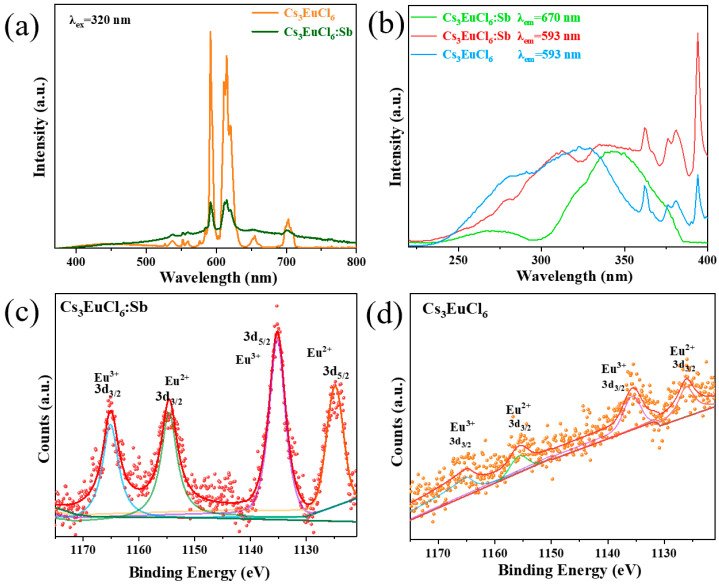
(**a**) PL spectra of Sb^3+^-doped and undoped Cs_3_EuCl_6_ NCs. (**b**) PLE spectra of Cs_3_EuCl_6_:Sb^3+^ NCs at 593 nm and 670 nm, and Cs_3_EuCl_6_ NCs at 593nm. (**c**,**d**) XPS spectra of Cs_3_EuCl_6_:Sb^3+^ (red dots) and Cs_3_EuCl_6_ (orange dots) NCs, respectively. (The blue line is Eu^3+^ 3d_3/2_, the green line is Eu^2+^ 3d_3/2_, the purple line is Eu^3+^ 3d_5/2_ and the yellow line is Eu^2+^ 3d_5/2_).

For Cs_3_EuCl_6_, the PL spectrum exhibits several sharp narrow-band emissions at 538, 593, 612, 650, and 703 nm, which are assigned to the intrinsic 4f-4f transitions of Eu^3+^ [38]. In addition, a weak broadband emission centered at 450 nm is observed, which may originates from Eu^2+^ [39]. After Sb^3+^ doping, the broadband emission at 450 nm remains unchanged, while a new broadband emission centered at 550 nm emerges, accompanied by a pronounced reduction in the Eu^3+^ narrow-band emissions. The phenomenon of luminescence is different from that of Sb^3+^-doped Cs_3_LnCl_6_ (Ln = Tb^3+^, Er^3+^, Yb^3+^, Ho^3+^, Sm^3+^) mentioned above. It is deduced that the Eu^3+^ emission in Cs_3_EuCl_6_:Sb^3+^ does not originate from energy transfer from STEs. To verify this assumption, the excitation spectra of the intrinsic Eu^3+^ narrow-band emission at 593 nm and the STE-related broadband emission at 670 nm in Cs_3_EuCl_6_:Sb^3+^ were recorded, as shown in Figure 4b. The excitation spectrum of the broadband emission is consistent with that of Cs_3_LaCl_6_:Sb^3+^, whereas the excitation monitored at 593 nm consists of two distinct components: (i) a broad feature in the ultraviolet region (220–360 nm), attributed to the ^1^S_0_-^1^P_1_ transition of the [SbCl_6_]^3−^ octahedra and the charge-transfer band (CTB) of the [EuCl_6_]^3−^ octahedra, indicating that the 593 nm emission originates from both Eu^3+^ characteristic emission and STE-related emission [40]; and (ii) several sharp peaks between 360 and 400 nm, corresponding to the intrinsic 4f-4f transitions of Eu^3+^. Consequently, when the excitation wavelength is within 300–360 nm, the Eu^3+^ emission intensity increases as the excitation wavelength decreases, while the STE emission intensity decreases. In contrast, at longer excitation wavelengths, a broadband emission centered at 450 nm becomes prominent, which can be attributed to Eu^2+^ emission, while the Eu^3+^ intrinsic emission remains relatively weak (Figure S11a). Further insights are obtained from XPS analysis, as shown in Figure 4c,d. The results confirm the coexistence of Eu^2+^ and Eu^3+^ in Cs_3_EuCl_6_, with atomic ratios of 29.60% and 70.40%, respectively, supporting the assignment of the 450 nm broadband emission to the 4f-5d transition of Eu^2+^ [41]. In contrast, Cs_3_EuCl_6_:Sb^3+^ shows a higher Eu^2+^ content of 54.25% and a reduced Eu^3+^ fraction of 45.75%, indicating that Sb^3+^ doping reduces the content of Eu^3+^, which accounts for the weakening of Eu^3+^ intrinsic emission [42]. Fluorescence decay measurements further verify the distinct origins of the 450 nm and 593 nm emissions, as illustrated in Figure S11b. In Cs_3_EuCl_6_, the 593 nm emission exhibits a long lifetime of 2.344 ms, consistent with the f-f transitions of Eu^3+^, whereas the 450 nm emission shows a lifetime of 4.960 ns (Figure S11c), in good agreement with the reported fluorescence lifetimes of Eu^2+^. For Cs_3_EuCl_6_:Sb^3+^, the increased Eu^2+^ content enhances Eu^2+^-Eu^2+^ interactions, leading to a shortened lifetime of 3.587 ns at 450 nm. Therefore, Eu^3+^, Eu^2+^, and STE emissions coexist in Cs_3_EuCl_6_:Sb^3+^, and no energy transfer occurs among them. This behavior is consistent with that observed in Cs_3_InCl_6_:Sb^3+^/Eu^3+^ [40].

**Figure 5:**
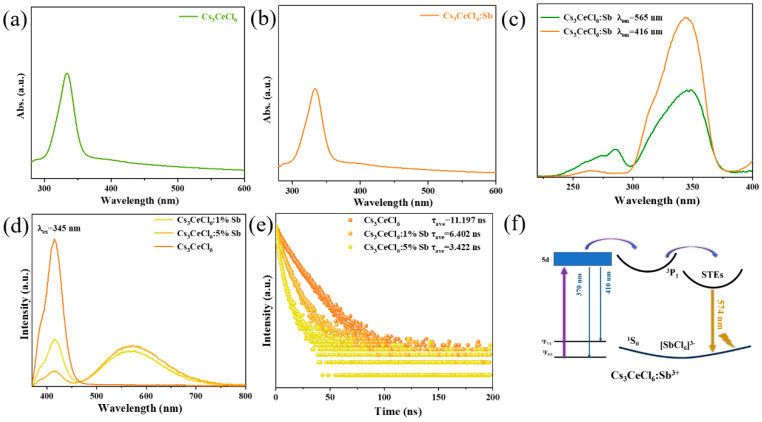
Absorption spectra of (**a**) Cs_3_CeCl_6_ and (**b**) Cs_3_CeCl_6_:Sb^3+^ NCs. (**c**) PLE spectra of Cs_3_CeCl_6_:Sb^3+^ NCs monitored at 565 nm and 416 nm. (**d**) PL spectra of Cs_3_CeCl_6_ NCs doped with different Sb^3+^ content. (**e**) PL decay curves of Cs_3_CeCl_6_ NCs doped with different Sb^3+^ content. (**f**) Schematic diagram of the luminescence mechanism of Cs_3_CeCl_6_:Sb^3+^ NCs.

For Cs_3_CeCl_6_:Sb^3+^, the luminescence mechanism is distinct from those of Cs_3_LnCl_6_:Sb^3+^ (Ln = Tb^3+^, Er^3+^, Yb^3+^, Ho^3+^, Sm^3+^, Eu^3+^) perovskites discussed above. As shown in Figure 5a, the absorption spectrum of Cs_3_CeCl_6_ exhibits a broadband in the range of 300–400 nm, which can be ascribed to the 4f-5d electronic transitions of Ce^3+^ [43]. Upon Sb^3+^ doping, the absorption edge remains essentially unchanged (Figure 5b). It is deduced that the luminescence source of Cs_3_CeCl_6_:Sb^3+^ is different from that of Cs_3_LaCl_6_:Sb^3+^. Under 345 nm excitation, the emission spectrum of Cs_3_CeCl_6_:Sb^3+^ exhibits not only a broadband centered around 550 nm but also an additional broad emission in the 370–450 nm region, which is attributed to the 5d-4f transition of Ce^3+^ [44]. To clarify the luminescence mechanism, the excitation spectra of Cs_3_CeCl_6_:Sb^3+^ monitored at 416 nm and 565 nm emission peaks were first measured. As shown in Figure 5c, the two excitation profiles exhibit nearly identical shapes, indicating that the two emissions originate from the same source. Based on these results, the 550 nm broadband is reasonably assigned to energy transfer involving Ce^3+^. To clarify this, Cs_3_CeCl_6_ doped with different concentrations of Sb^3+^ were prepared, and the PL spectra and decay curves were monitored. As shown in Figure 5d, the spectrum of Cs_3_CeCl_6_ exhibits emission peaks at 370 and 416 nm under 345 nm excitation, which originate from the 5d-^2^F_5/2_ and 5d-^2^F_7/2_ transitions of Ce^3+^ ions [45]. Upon Sb^3+^ incorporation, an additional broad emission band centered at 574 nm emerges, indicating that this band is induced by the introduction of Sb^3+^. With further increase in Sb^3+^ concentration, the emission intensity of Ce^3+^ gradually decreases, while the broadband at 574 nm becomes more pronounced. The fluorescence decay curves monitored at 416 nm are presented in Figure 5e. The fitting results reveal that the lifetime decreases from 11.20 ns to 3.42 ns as the Sb^3+^ content increases, confirming that the broadband emission at 574 nm originates from energy transfer from Ce^3+^ ions. To better illustrate the underlying photophysical processes, a schematic diagram of the emission mechanism of Cs_3_CeCl_6_:Sb^3+^ is shown in Figure 5f. Under 345 nm excitation, electrons of Ce^3+^ are excited to the 5d state. The electrons then undergo radiative transitions to the ^2^F_5/2_ and ^2^F_7/2_ levels, giving rise to blue-violet emissions at 370 and 416 nm. Meanwhile, because the excited state of Ce^3+^ is close to the excited state of [SbCl_6_]^3−^ octahedron, the effective energy transfer from the 5d level to the ^3^P_1_ level can occur, followed by the transfer to the STE, which finally transitions to the ground state and generates the broadband emission.

In the field of optical anti-counterfeiting, variation in emission spectra from specific luminescent materials can serve as a unique and secure identifier. Although some studies have attempted to achieve red emission using simpler Eu-based hosts, such as Eu^3+^-doped Cs_2_NaInCl_6_, Cs_2_NaYCl_6_, and Cs_2_AgInCl_6_ [46,47,48], these systems generally provide only a single red emission channel and lack the multifunctionality required for advanced anti-counterfeiting applications. Based on this, a dual-mode anti-counterfeiting strategy was developed using Sb^3+^-doped Cs_3_Tb/EuCl_6_ NCs, which combine color-tunable visible emission and intensity-dependent spectral coding. When excited by commercial ultraviolet light, these materials display distinct emission colors that can be directly distinguished by the naked eye. Moreover, the relative intensities of the characteristic Tb^3+^ and Eu^3+^ emission peaks vary with the ratio of Tb^3+^ and Eu^3+^, allowing for spectral encoding based on both peak position and intensity—a feature that requires specialized spectroscopic equipment to analyze and is extremely difficult to replicate.

**Figure 6:**
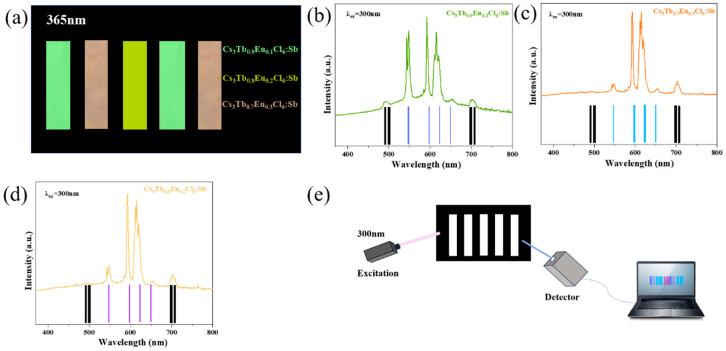
(**a**) Patterns made of Cs_3_Tb_0.9_Eu_0.1_Cl_6_:1%Sb^3+^, Cs_3_Tb_0.8_Eu_0.2_Cl_6_:1%Sb^3+^, and Cs_3_Tb_0.7_Eu_0.3_Cl_6_:1%Sb^3+^ NCs emit orange, yellow, and green under the excitation at 365 nm ultraviolet light. (**b**–**d**) PL spectra of Cs_3_Tb_0.9_Eu_0.1_Cl_6_:1%Sb^3+^, Cs_3_Tb_0.8_Eu_0.2_Cl_6_:1%Sb^3+^, and Cs_3_Tb_0.7_Eu_0.3_Cl_6_:1%Sb^3+^ NCs under the excitation at 300 nm and their corresponding sub-barcodes. (**e**) Diagram of the detection experimental setup.

To demonstrate this concept, Cs_3_Tb_0.9_Eu_0.1_Cl_6_:1%Sb^3+^, Cs_3_Tb_0.8_Eu_0.2_Cl_6_:1%Sb^3+^, and Cs_3_Tb_0.7_Eu_0.3_Cl_6_:1%Sb^3+^ NCs were synthesized. The corresponding nanoparticle powders were arranged in a barcode-like pattern to achieve visually encoded emission. Under excitation with a 365 nm commercial UV lamp, distinct green, yellow, and orange luminescence can be observed, as shown in Figure 6a. When excited at 300 nm, the intensity ratios of the Tb^3+^ and Eu^3+^ emission peaks change correspondingly, and each composition produced a characteristic spectrum that could be encoded into a unique optical barcode (Figure 6b–d). As illustrated in Figure 6e, when the barcode pattern is irradiated with a 300 nm light source, a detector collects the emitted spectra and translates them into digital barcode information through spectral analysis [49,50].

This dual-mode strategy—combining visible color recognition and spectral encoding—offers a reliable, multi-level, and hard-to-forge optical anti-counterfeiting approach. The results demonstrate that the composition-dependent luminescence properties of Sb^3+^-doped Cs_3_Tb/EuCl_6_ materials hold strong potential for advanced information encryption and high-security labeling applications. For instance, a prescription drug package can be coated with a barcode fabricated from the anti-counterfeiting material. Prior to scanning, consumers can visually confirm the authenticity of the package by observing the distinct luminescent barcode under a 365 nm light source, ensuring that the regulatory label has not been replaced or tampered with. Detailed product information is subsequently retrieved using an optical reader capable of decoding the spectral signals.

## 4. Conclusions

In summary, a series of Sb^3+^-doped Cs_3_LnCl_6_ (Ln: Yb, La, Eu, Ho, Ce, Er, Tb, Sm, Y) nanocrystals were successfully synthesized to explore the regulatory effects of Sb^3+^ on the structural and optical properties of rare-earth-based halide perovskites. All samples maintain a highly crystalline 0D monoclinic phase with isolated [LnCl_6_]^3−^ octahedra, ensuring strong exciton confinement and structural stability. The introduction of Sb^3+^ not only induces intense broadband STE emission through Jahn–Teller-distorted [SbCl_6_]^3−^ units but also establishes efficient STE-Ln^3+^ energy transfer channels. This results in enhanced luminescence intensity, improved spectral tunability, bandgap narrowing, and higher photon absorption efficiency. Moreover, the luminescence behavior of different lanthanide hosts demonstrates diverse mechanisms: La-based systems show STE-dominated emission, while Tb-, Er-, Yb-, Ho-, and Sm-based materials exhibit STE-mediated f-f emission enhancement. In contrast, Eu- and Ce-based hosts reveal unique pathways involving Eu^2+^/Eu^3+^ valence conversion and Ce^3+^ → STE energy transfer. The composition-dependent emissions of Sb^3+^-doped Cs_3_Tb/EuCl_6_ further enable dual-mode color and spectral encoding, providing a new concept for optical anti-counterfeiting. This study demonstrates that Sb^3+^ serves as an effective multifunctional dopant for tailoring electronic structures, promoting energy transfer, and expanding emission tunability in rare-earth halide systems. The findings provide valuable guidance for designing next-generation luminescent and optoelectronic materials with high efficiency, stability, and multifunctionality.

## Data Availability

Data is contained within the article or Supplementary Materials.

## Supplementary Materials

The following supporting information can be downloaded at: https://www.mdpi.com/article/10.3390/nano15231790/s1.
