# Supplementary material for: Tunable Luminescence in Sb3+-Doped Cs3LnCl6 Perovskites for Wide-Coverage Emission and Anti-Counterfeiting Applications
Source: Nanomaterials (Basel). 2025 Nov 27;15(23):1790. doi: 10.3390/nano15231790 (PMC12693347; doi:10.3390/nano15231790)
Supplement: Supplementary file 1 [file nanomaterials-15-01790-s001.zip › nanomaterials-3967025-supplementary.pdf]

Tunable Luminescence in Sb^{3+}-Doped Cs_3LnCl_6 Perovskites for Wide-coverage Emission and Anti-Counterfeiting Applications

Lianao Zhang[1], Le Chen[1], Sai Xu[1,*], Yongze Cao[1], Xizhen Zhang[1], Hongquan Yu[1], Yuefeng Gao[2,*], Baojiu Chen[1]

[1]School of Science, Dalian Maritime University, Dalian 116026, People's Republic of China

[2]Marine Engineering College, Dalian Maritime University, Dalian 116026, People's Republic of China

*Corresponding authors

E-mail address: gaoyuefeng@dlmu.edu.cn (Y. Gao), xsjlu@126.com (S. Xu)

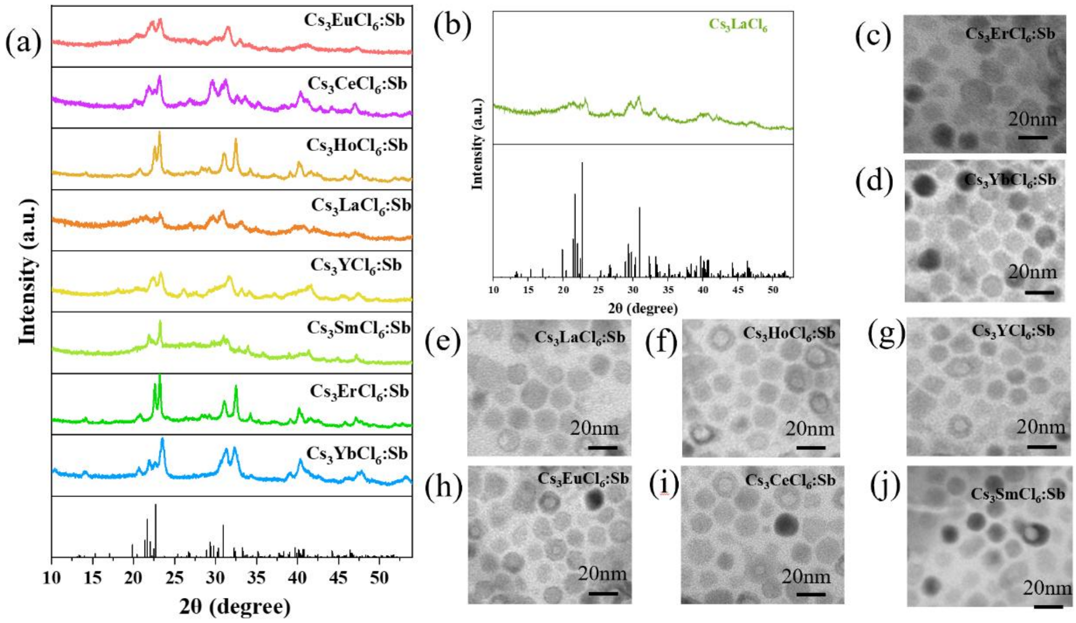

Figure S1 (a) XRD patterns (b) XRD pattern of Cs_3LaCl_6 (c-j) TEM images of $Cs_3LnCl_6:Sb^{3+}$ NCs (Ln: Yb, La, Eu, Ho, Ce, Er, Sm, Y) and Cs_3LaCl_6

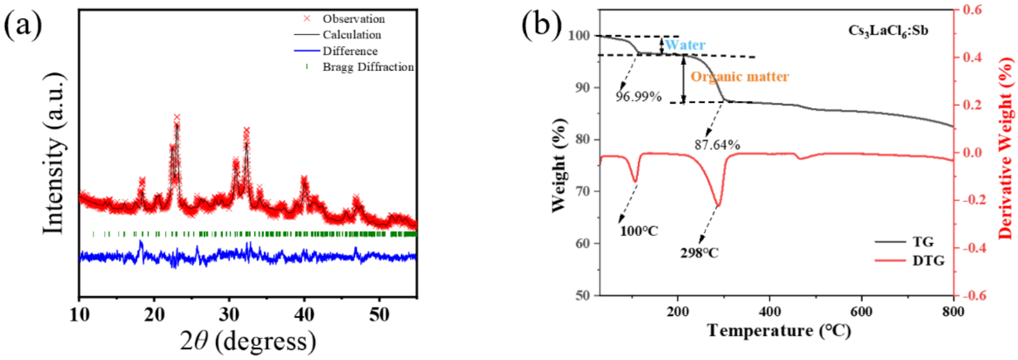

Figure S2 (a)XRD Rietveld refinement of $Cs_3EuCl_6:Sb^{3+}$ (b) TG curves of $Cs_3LaCl_6:Sb^{3+}$

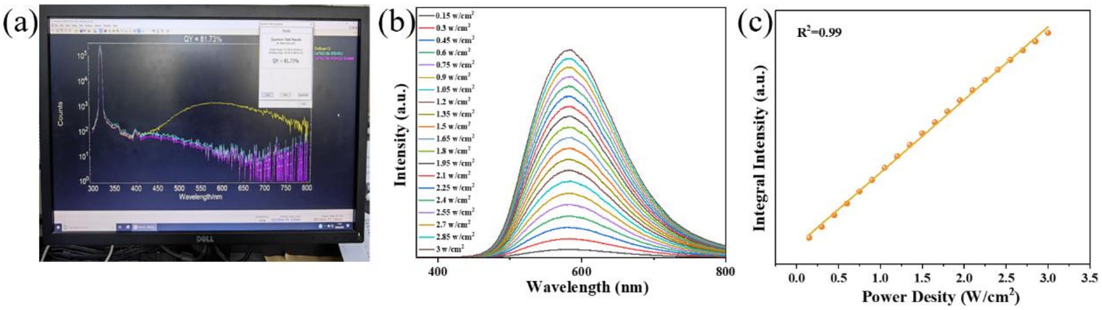

Figure S3 (a) PLQY test result of $Cs_3LaCl_6:Sb^{3+}$ NCs (b) PL spectra of $Cs_3LaCl_6:Sb^{3+}$ NCs under different power densities (c) Linear fitting of power density and emission intensity of $Cs_3LaCl_6:Sb^{3+}$ NCs

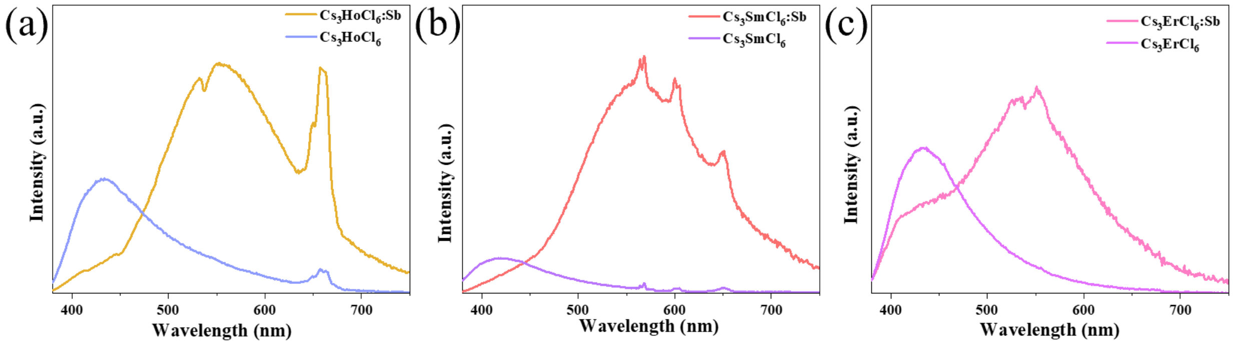

Figure S4 (a-c) PL spectra comparison of $Cs_3LnCl_6:Sb^{3+}$ and Cs_3LnCl_6 (Ln:Ho,Sm,Er) NCs excited by 320 nm.

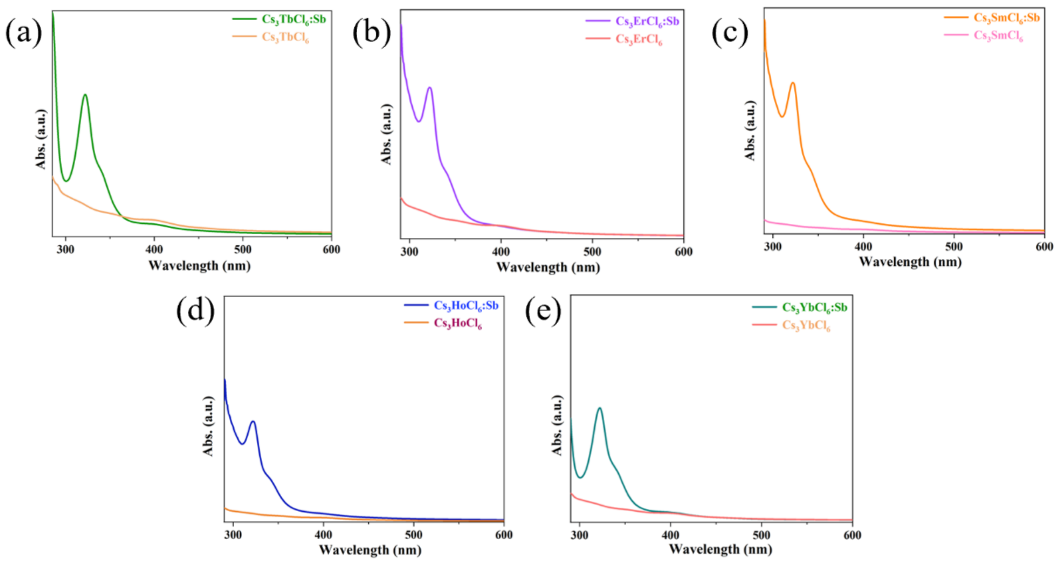

Figure S5 (a-e) Absorption spectra comparison of $Cs_3LnCl_6:Sb^{3+}$ and Cs_3LnCl_6 (Ln: Tb,Yb,Ho,Ee,Sm) NCs

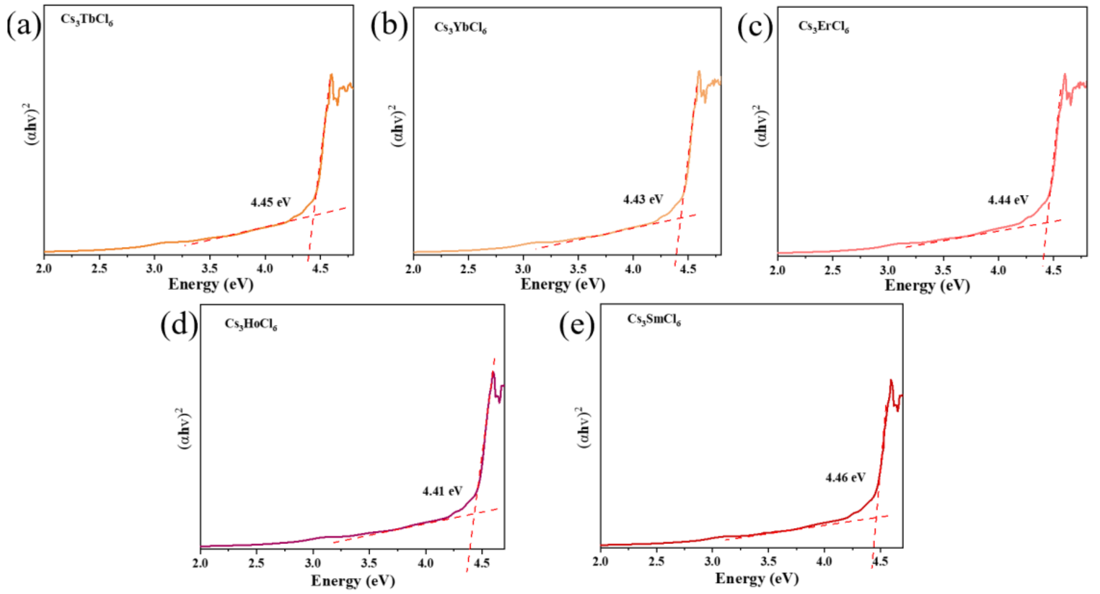

Figure S6 (a-e) Tauc plots of Cs_3LnCl_6 (Ln:Yb,Er,Ho,Sm,Y) NCs

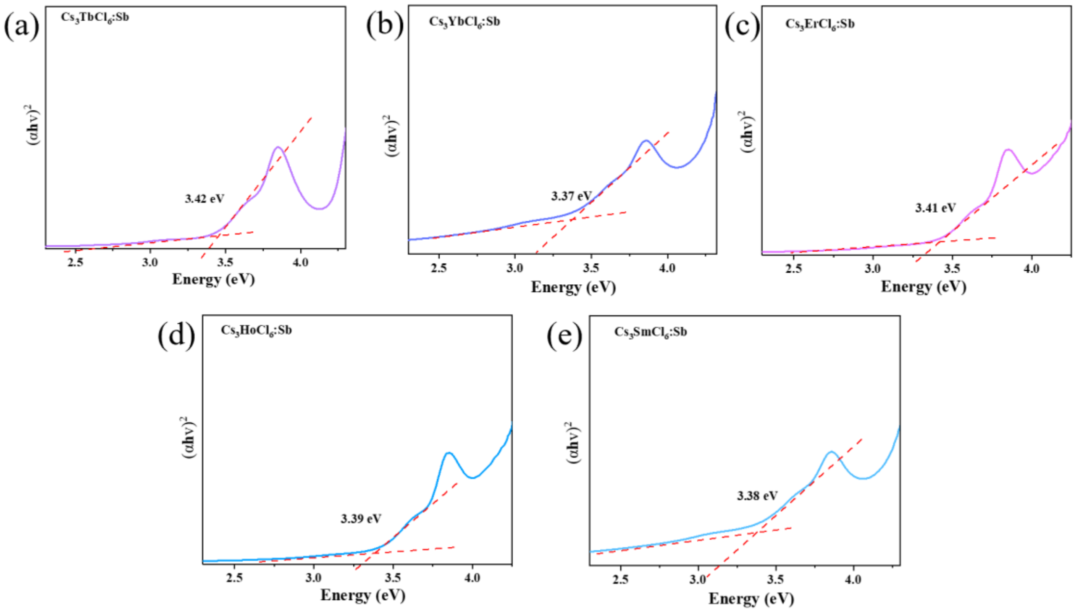

Figure S7 (a-e) Tauc plots of $Cs_3LnCl_6:Sb^{3+}$ (Ln:Yb,Er,Ho,Sm,Y) NCs

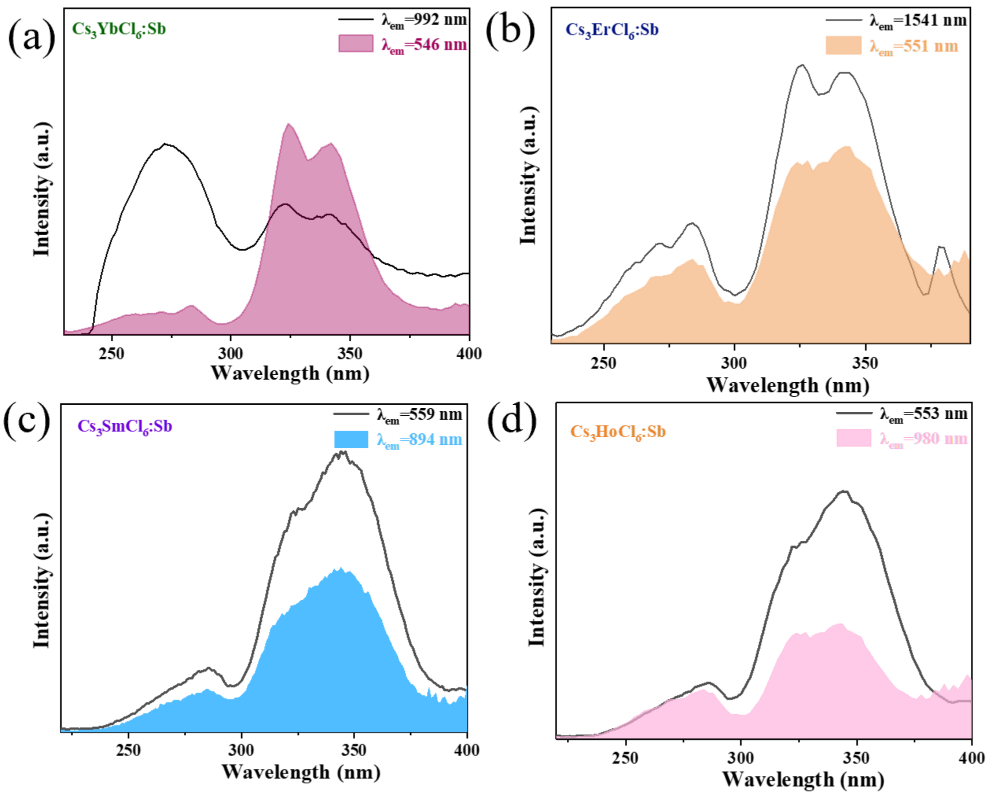

Figure S8 (a-d) The excitation spectra of $Cs_3LnCl_6:Sb^{3+}$ (Ln:Yb,Er,Ho,Sm)NCs

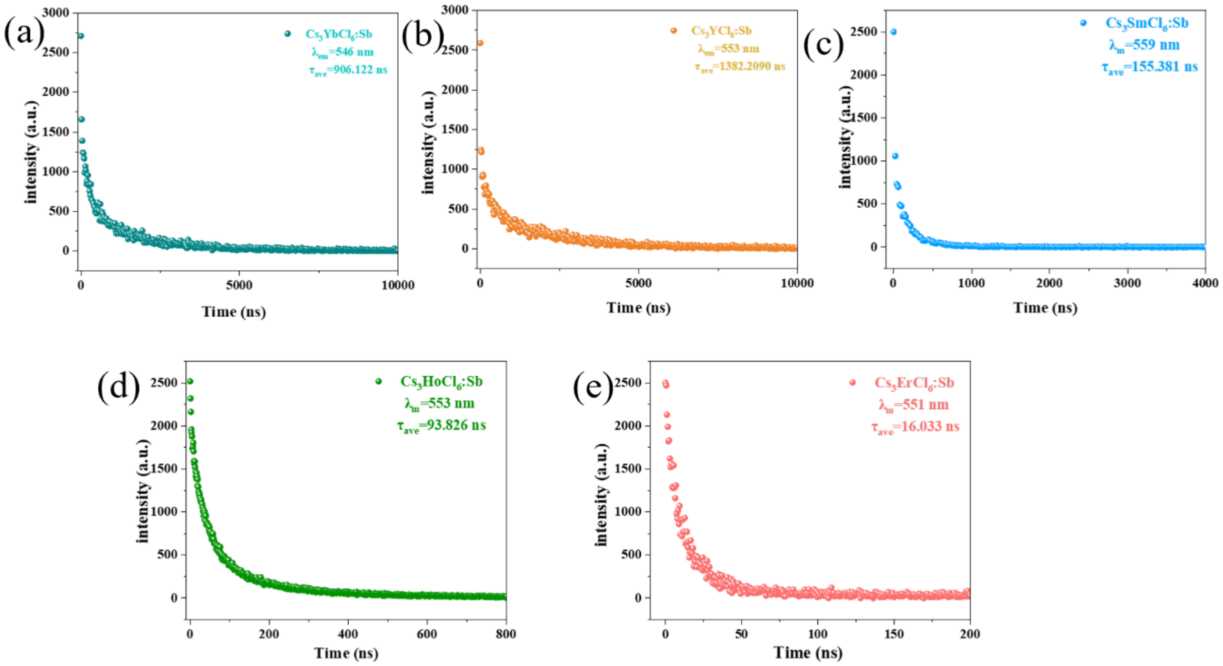

Figure S9 (a-e) PL decay curves of $Cs_3LnCl_6:Sb^{3+}$ (Ln:Yb,Er,Ho,Sm,Y) NCs

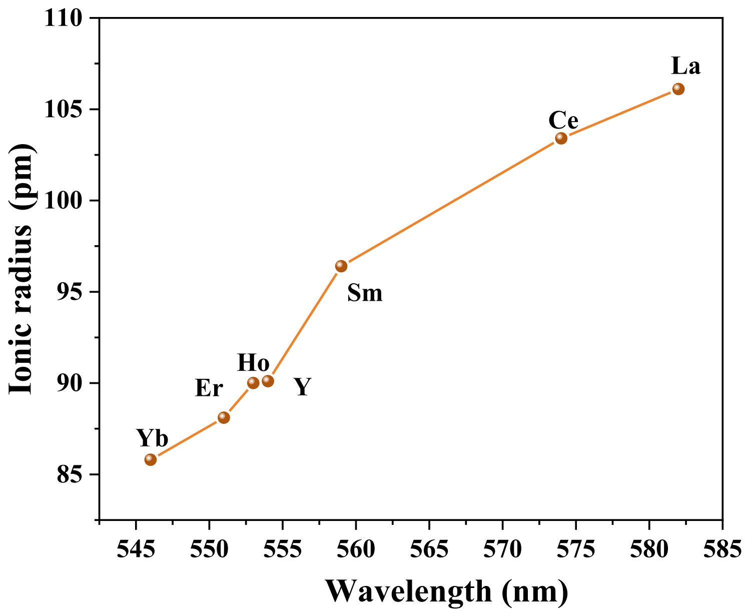

Figure S10 Relationship diagram between luminescent center and ion radiu in

$Cs_3LnCl_6:Sb^{3+}$ (Ln:Yb,Er,Ho,Sm,Y, Ce, La) NCs

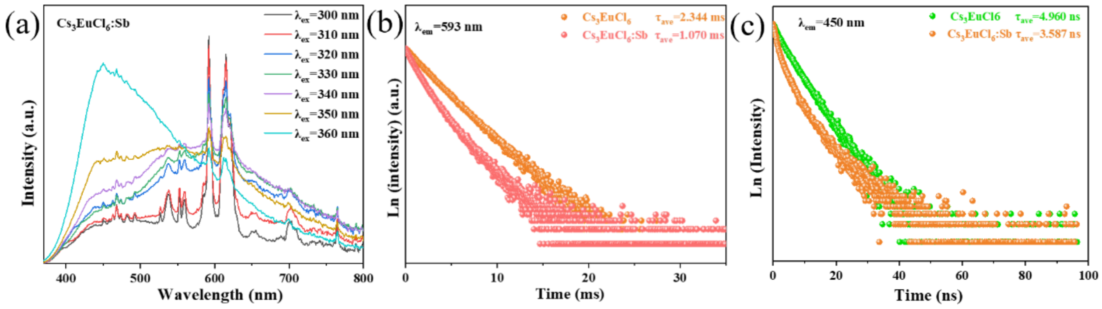

Figure S11 (a) Emission spectra of Cs_3EuCl_6:Sb^{3+} under different excitation wavelengths (b) PL decay curves of Sb^{3+}-doped and undoped Cs_3EuCl_6 NCs monitored at 593nm (c) The PL decay curves of Sb^{3+}-doped and undoped Cs_3EuCl_6 NCs monitored at 450nm

Table S1. Rietveld refinement parameters for $Cs_3EuCl_6:Sb^{3+}$.

Space group	C2/c
R_p	4.82%
R_{wp}	6.18%
R_{exp}	4.03%
χ^2	2.35
V (Å^3)	2742.721
a (Å)	26.76791
b (Å)	7.74894
c (Å)	13.41643

Table S2. Fitted lifetimes of Cs_3LaCl_6:x%Sb^{3+} NCs monitored at 580 nm under the excitation of 320 nm.

x	τ_1 (μs)	A_1 (%)	τ_2 (μs)	A_2 (%)	τ_{ave} (μs)
0.5	0.012	83.645	2.681	16.355	2.620
1	0.011	80.099	2.658	19.901	2.614
2	0.017	69.032	2.700	30.968	2.662
5	0.049	58.968	2.260	41.032	2.193

Table S3. Energy transfer between Cs_3LnCl_6 and Sb^{3+}

sample	energy transition
$Cs_3LaCl_6:Sb^{3+}$	STEs
$Cs_3YCl_6:Sb^{3+}$	STEs
$Cs_3TbCl_6:Sb^{3+}$	STEs→$^5D_4$
$Cs_3ErCl_6:Sb^{3+}$	STEs→$^4S_{3/2}$
$Cs_3SmCl_6:Sb^{3+}$	STEs→$^4G_{5/2}$
$Cs_3HoCl_6:Sb^{3+}$	STEs→$^4S_2$
$Cs_3YbCl_6:Sb^{3+}$	STEs→$^5F_{5/2}$
$Cs_3CeCl_6:Sb^{3+}$	5d→STEs
